# An outbreak of a rare Shiga-toxin-producing *Escherichia coli* serotype (O117:H7) among men who have sex with men

**DOI:** 10.1099/mgen.0.000181

**Published:** 2018-05-21

**Authors:** Kate S. Baker, Timothy J. Dallman, Nicholas R. Thomson, Claire Jenkins

**Affiliations:** ^1^​ Institute for Integrative Biology, University of Liverpool, Liverpool, UK; ^2^​ Public Health England, Colindale, London, UK; ^3^​ Wellcome Trust Sanger Institute, Hinxton, UK

**Keywords:** STEC, MSM, antimicrobial resistance, outbreak

## Abstract

Sexually transmissible enteric infections (STEIs) are commonly associated with transmission among men who have sex with men (MSM). In the past decade, the UK has experienced multiple parallel STEI emergences in MSM caused by a range of bacterial species of the genus *Shigella*, and an outbreak of an uncommon serotype (O117 : H7) of Shiga-toxin-producing *Escherichia coli* (STEC). Here, we used microbial genomics on 6 outbreak and 30 sporadic STEC O117 : H7 isolates to explore the origins and pathogenic drivers of the STEC O117 : H7 emergence in MSM. Using genomic epidemiology, we found that the STEC O117 : H7 outbreak lineage was potentially imported from Latin America and likely continues to circulate both in the UK MSM population and in Latin America. We found genomic relationships consistent with existing symptomatic evidence for chronic infection with this STEC serotype. Comparative genomic analysis indicated the existence of a novel Shiga toxin 1-encoding prophage in the outbreak isolates, and evidence of horizontal gene exchange among the STEC O117 : H7 outbreak lineage and other enteric pathogens. There was no evidence of increased virulence in the outbreak strains relative to contextual isolates, but the outbreak lineage was associated with azithromycin resistance. Comparing these findings with similar genomic investigations of emerging MSM-associated *Shigella* in the UK highlighted many parallels, the most striking of which was the importance of the azithromycin phenotype for STEI emergence in this patient group.

## Data Summary

Three supplementary figures and two supplementary tables are available with the online version of this article. All supporting data and protocols have been provided within these supplementary files or the main article.

**Outcome**

This study investigates, using genomics with contextual data, the emerging problem of sexually transmissible enteric infections (STEIs) among men who have sex with men (MSM). It demonstrates that relatively uncommon enteric pathogens can emerge as domestically transmitting epidemics in this unique patient group associated with antimicrobial resistance, in the absence of important virulence factors. Furthermore, the parallels drawn between this study and similar studies on other emerging STEIs in the same MSM community indicates how microbial genomic studies build on each other to reveal common drivers of disease processes.

## Introduction

Certain enteric pathogens traditionally associated with food-borne or person–person spread can also be transmitted sexually. Outbreaks of such sexually transmissible enteric infections (STEIs) are frequently associated with transmission in men who have sex with men (MSM) in whom oro-anal and genital-anal contact is common. The wide variety of STEIs includes bacteria of the genera *Shigella*, *Escherichia*, *Salmonella* and *Campylobacter*, and the parasites *Giardia lamblia* and *Entamoeba histolytica* [1], and a number of these have emerged recently in the UK in parallel with an increase in traditional sexually transmitted infections (STIs) [2]. Specifically, there has been an intensification of MSM-associated diarrhoeal disease caused by diverse bacteria of the genus *Shigella* since 2009 [3], which genomic studies have revealed is associated with antimicrobial resistance (AMR) [4–6]. In addition to these prolonged and overlapping shigellosis epidemics, a domestically transmitting outbreak of a Shiga-toxin-producing *Escherichia coli* (STEC) O117 : H7 was observed in 2014 in the UK MSM community [7]. STEC are an important cause of gastrointestinal symptoms worldwide, and certain virulence determinants, such as the locus of enterocyte effacement (LEE) pathogenicity island (PAI) and the Shiga toxin (Stx) subtype 2a are associated with epidemic emergence, and the elaboration of more severe disease syndromes such as haemolytic-uraemic syndrome [8, 9].

In the UK, STEC O117 : H7 is a rare serotype, often misidentified as *Shigella sonnei* at local laboratories owing to their biochemical similarities and is typically isolated from patients reporting recent travel abroad [10, 11]. Hence, a domestically transmitting epidemic of this uncommon serotype was unexpected. Full epidemiological and clinical features of the STEC O117 : H7 outbreak are reported elsewhere [7], but briefly, between November 2013 and August 2014, an outbreak of nine patients occurred. These patients reported diarrhoeal and other symptoms (e.g. stomach cramps, fatigue) that were often prolonged (one patient reported symptoms of 1 month duration prior to seeking care). Eight of the nine patients were MSM who contracted their infection in the UK, many of whom had risk factors in common with those for other STEIs, including the UK MSM *Shigella* epidemics (i.e. human immunodeficiency virus infection, high numbers of sexual partners) [3, 7, 12].

To investigate the emergence of this rare STEC serotype in MSM, and explore parallels and contrasts with the concurrent *Shigella* epidemics, we performed whole-genome sequence analysis (WGSA) on six isolates from the 2014 STEC O117 : H7 outbreak, and a further 30 contextual STEC O117 : H7 isolates collected between 2006 and 2017.

## Methods

### Isolates and sequencing by synthesis (Illumina sequencing)

In total, Illumina sequence data from 36 isolates of STEC O117 : H7 submitted to, and sequenced by, the Gastrointestinal Bacteria Reference Unit (GBRU) at Public Health England (PHE) were examined in this study (Table 1). All available sequencing data from STEC O117 : H7 isolates were included in this study. Specifically, this included data from: isolates sequenced as part of our work published in 2013 [10] (*n*=5) and other investigations (*n*=5); isolates sequenced as part of the outbreak investigation (*n*=6); and all O117 : H7 isolated since the implementation of routine WGSA surveillance at PHE (*n*=20). Basic patient epidemiological data from sample submission forms (sex, age, history of recent travel if noted) were also retrieved. Whole-genome sequencing was performed at PHE using Nextera XT DNA sample preparation kits and sequencing on an Illumina HiSeq 2500 instrument [13]. Sequence data quality was checked using fastqc (v 0.11.4), and adapter sequences and 5′ and 3′ ends (14 and 3 bp, respectively) were trimmed to remove sequence per base content biases using trim galore v. 0.4.5 (cutadapt v 1.13). Existing sequencing data was retrieved from the Short Read Archive, where novel data was also deposited, under bioproject PRJNA315192 (further accession numbers are given in Table S1, available with the online version of this article). Draft genome assemblies were generated from quality-trimmed data using spades v. 3.11.0 [14], improved using a standalone assembly improvement pipeline [15] based on scaffolding with sspace [16] and gap filling using GapFill [17], and annotated using prokka v 1.11 [18].

**Table 1.** Isolates of STEC O117 : H7

Source	Years	Reference	Isolates (*n*)
Routine WGS surveillance	2014–2017	This paper	20
2014 MSM-outbreak investigation	2013–2014	This paper, epidemiology reported elsewhere [7]	6
Non-routine WGS surveillance	2006–2013	This paper and [10]	10

### Single molecule-real time (SM-RT) sequencing of two outbreak isolates

Complete (but unfinished) genomes were generated for two outbreak isolates (23168 and 23169, Table S1) through SM-RT sequencing (as in [6]). Briefly, genomic DNA (200 ng) was extracted using the MasterPure Complete DNA and RNA extraction kit, and sequenced on individual SM-RT cells on a Pacific Biosciences RSII system at the Wellcome Trust Sanger Institute (Hinxton, UK). Sequencing data were assembled using hgap v3 [19], circularized using Circlator v 1.1.3 20] and polished with Quiver v1 (as in [6]).

### Phylogenetic analysis

To infer a phylogenetic tree describing the evolutionary relationships among isolates, trimmed sequencing data were mapped against the complete genome of isolate 23169 using smalt (http://www.sanger.ac.uk/science/tools/smalt-0). For all isolates after mapping, >90 % of the genome was covered, with repeat mappings discarded (i.e. reads with multiple best mappings). Overall, between 88 and 95 % of reads for each isolate mapped with a mean depth of 20–90 times coverage (Table S1). Variants were called with Samtools [21] to generate a reference-based multiple sequence alignment of pseudogenomes. The region of the alignment relating to the bacterial chromosome (contiguous sequence 1 – see Results) was extracted and recombination removed using gubbins [22]. In total, 3694 non-recombinant variant sites extracted using *SNP*-*sites* [23] among the 36 isolates were used to infer a maximum-likelihood phylogeny using RAxML v 8.2.8 [24]. To facilitate discussion and investigation of the outbreak, the well-supported (bootstrap value of 100) most recent common ancestor of all six epidemiologically confirmed outbreak isolates was identified and named the ‘outbreak clade’. Visualizations were made using iToL [25].

### Genomic analysis

To determine the basic architecture of the genome, the identities of contiguous sequences from the complete genome assemblies of isolates 23168 and 23169 were compared with existing sequences and each other. Features common to both isolates were considered part of the basic genome architecture. For detection of PAIs, sequences for the LEE-PAI, locus of adhesion and autoaggregation (LAA) PAI and the subtilase-encoding PAI (SE-PAI) were downloaded from the National Center for Biotechnology Information (NCBI) database, using accession numbers AF022236, AFDQ01000026.1 (positions 385984–472336) and JQ9944271, respectively. Genome annotations and phast analysis [26] were used to identify the location of the Stx-encoding phage. blast [27] of the putative Stx-containing prophage region against the non-redundant NCBI nucleotide database was then used to identify the closest match (NCBI accession no. NC_028685.1), which was then used in comparative analysis. Pairwise comparison of phage sequences was determined using the emboss stretcher modification of the Needleman–Wunsch algorithm that allows larger sequences to be globally aligned [28]. For the detection of virulence and acquired AMR genes, draft and complete genomes were analysed on the ResFinder and VirulenceFinder web interfaces at the Centre for Genomic Epidemiology [29, 30], and the amino acids encoded at the following sites (known to alter fluoroquinolone susceptibility) were examined in each isolate: *gyrA* codons 83 and 87; *parC* codons 80, 84 and 129. Plasmid visualizations were made using the blast ring image generator [31].

### Azithromycin testing

Phenotypic testing of a subset of isolates for reduced susceptibility against azithromycin was performed using the Etest macromethod (bioMérieux), and statistical comparison of the frequency of the *mphA* gene occurrence in outbreak clade and non-outbreak clade isolates was calculated using a two-tailed Fisher’s exact test.

## Results

### Phylogenetic context

**Fig. 1.**
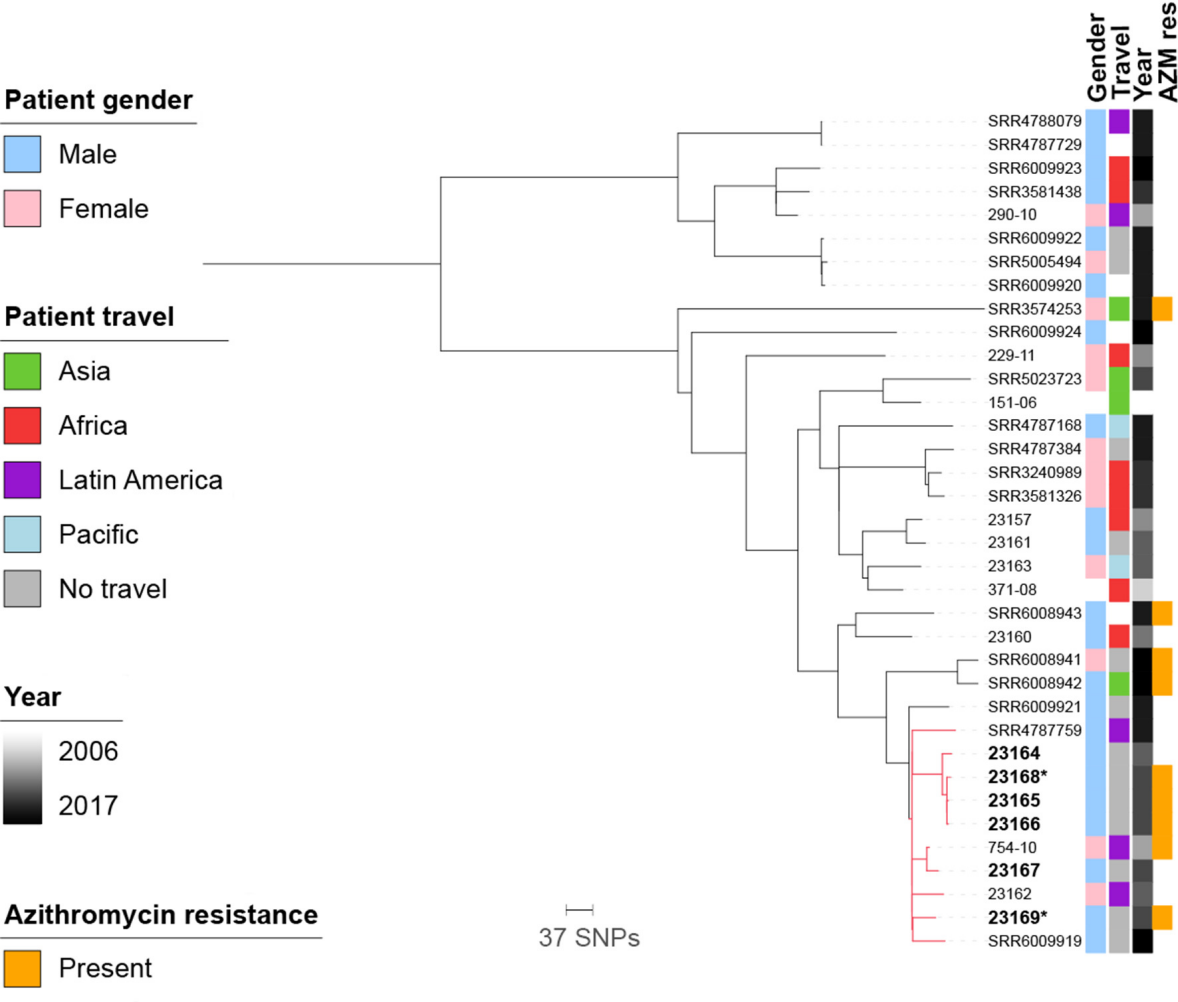
The mid-point rooted maximum-likelihood phylogenetic tree shows the evolutionary relationships among 36 STEC O117 : H7 isolates. Epidemiologically determined outbreak isolates are indicated in bold and the monophyletic outbreak clade is shown in red. The two outbreak isolates that underwent subsequent SM-RT sequencing are indicated by asterisks. The tracks adjacent to the tree trips show patient gender, travel history, year of isolation (heatmapped) and the presence of azithromycin (AZM)-resistance gene(s) according to the inlaid key. Missing data in the gender and travel columns are indicated in white.

To gain epidemiological insight into the outbreak of this rare STEC serotype, the evolutionary relationships among the isolates were combined with the patient epidemiological metadata. This revealed that the six epidemiologically confirmed outbreak isolates shared close genetic relationships with four non-epidemiologically investigated isolates contained within the monophyletic outbreak clade (Fig. 1). Of the ten outbreak clade isolates, eight were from men and two were from women. The four non-epidemiologically confirmed isolates in the outbreak clade comprised two samples from non-routine WGS surveillance taken in 2010 and 2013 from patients recently returned from Latin America, and two samples isolated much later in 2016 (also from a recently returned Latin American traveller) and 2017 (submitted in London from a 26-year-old male patient with no history of travel). Outside of the outbreak clade, there was also one patient from whom serially collected isolates were submitted, the first on the 26th of April 2016 after recent travel to Latin America (SRR4788079), and the second on the 8th of August 2016 with no travel history noted (SRR4787729). These two samples were phylogenetically adjacent and showed no single nucleotide polymorphisms (SNPs) between them.

### Genome architecture

**Fig. 2.**
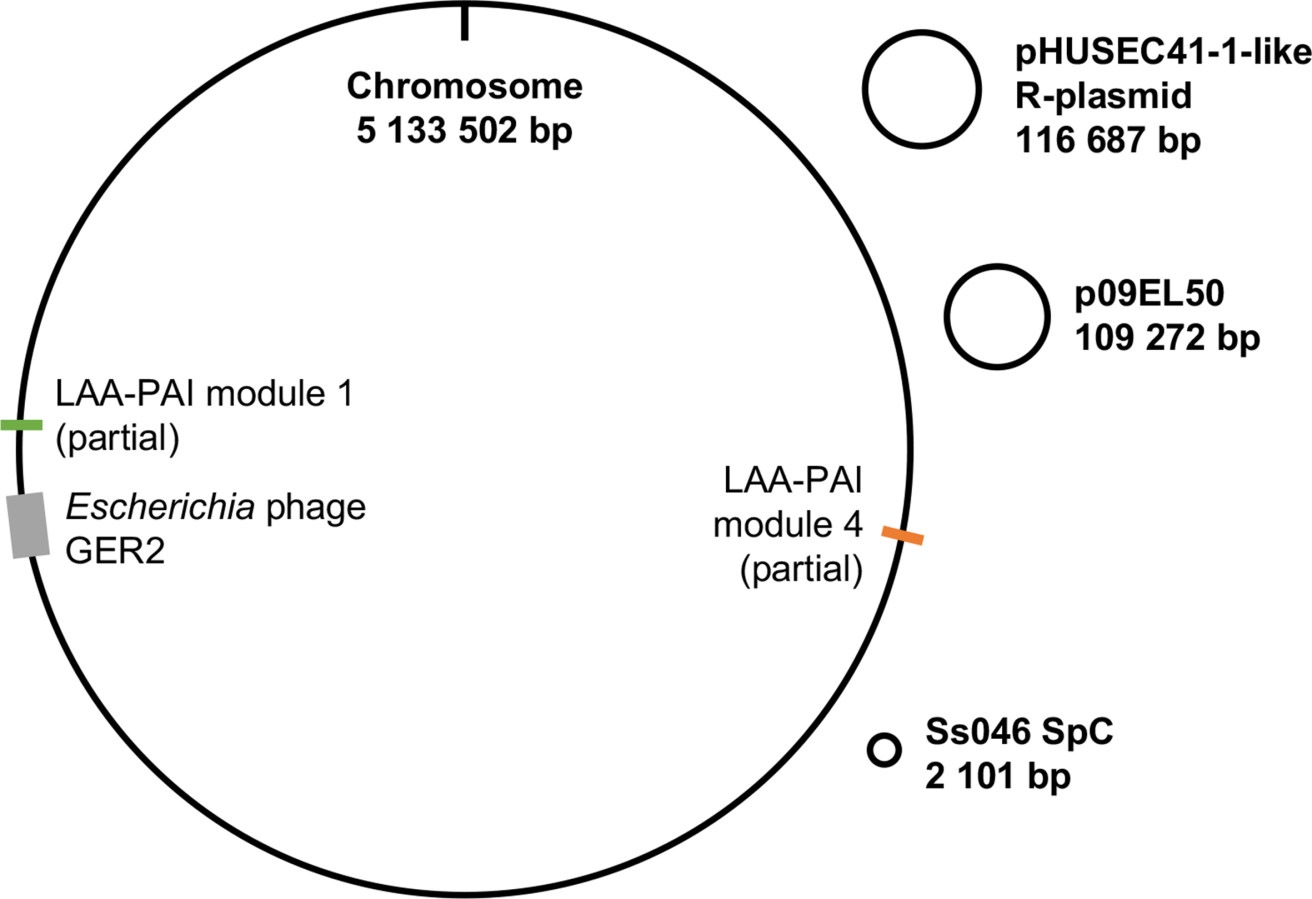
Genome schematic of the STEC O117 : H7 outbreak strain. The bacterial chromosome carries a novel Stx-encoding prophage and partial modules of the LAA-PAI. Plasmids in the strain include: a small cryptic plasmid (Ss046 SpC), a larger resistance plasmid (pHUSEC41-1-like R-plasmid) and another larger plasmid (p09EL50).

**Fig. 3.**
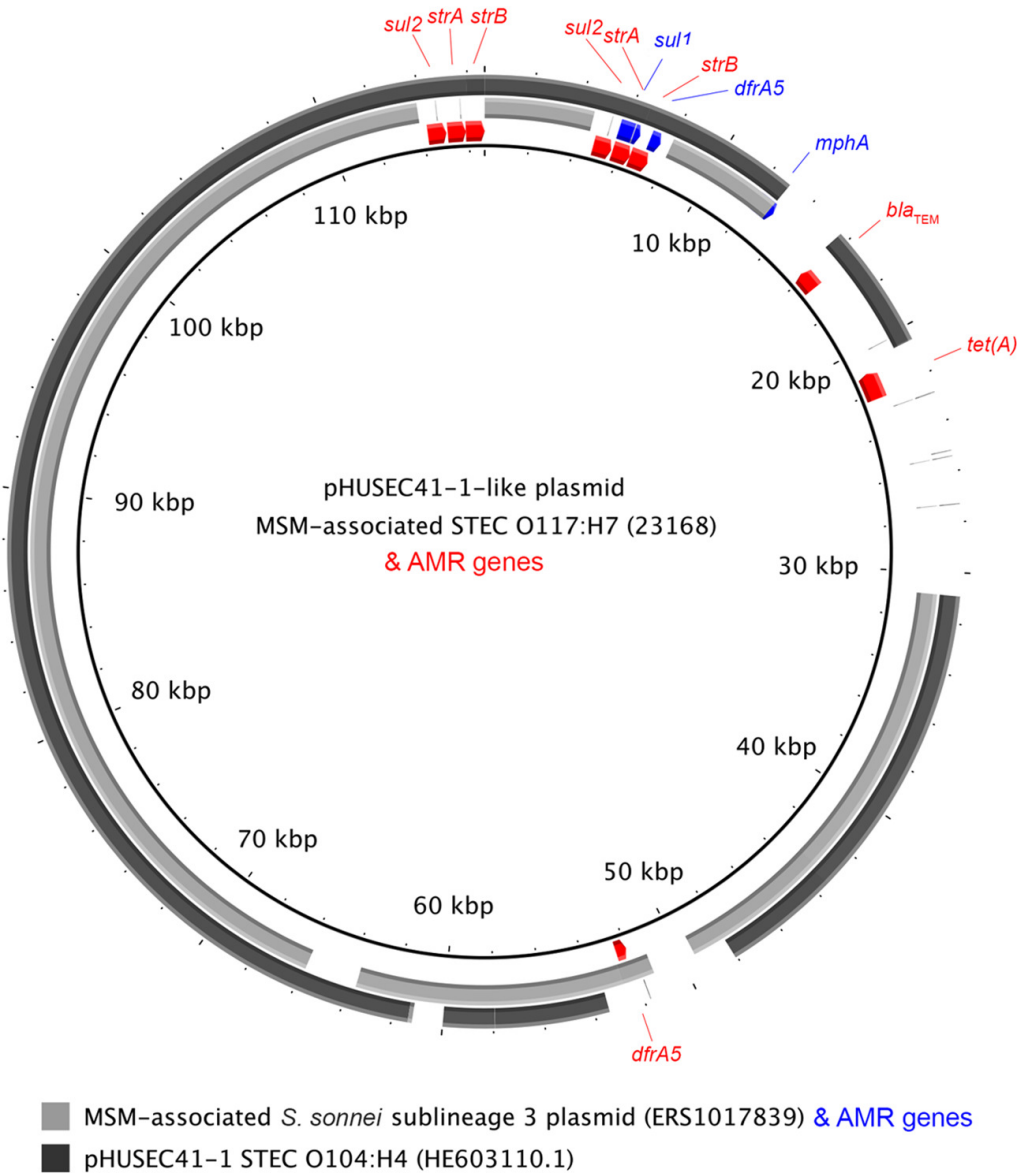
Plasmid comparative genomics. The pHUSEC41-1-like plasmid from the MSM-associated STEC O117 : H7 outbreak strain (isolate 23168) is shown centrally, with AMR genes on the plasmid annotated as red arrows in the adjacent ring. Regions of plasmid similarity (70–100 % with blast) to MSM-associated *S. sonnei* sublineage 3 plasmid (isolate ERS1017839) [6] and pHUSEC41-1 from STEC O104:H4 (NCBI accession no. HE603110.1) are shown in grey in the outer rings according to the inlaid key. Additional AMR genes carried on the MSM-associated *S. sonnei* sublineage 3 plasmid are shown in blue. AMR gene names are shown outermost.

To characterize the genome features of the strain responsible for the MSM-associated outbreak, complete genomes were generated for two outbreak isolates (isolates 23168 and 23169). These assembled into 12 and 15 contiguous sequences, respectively (Table S2). The two genomes were broadly syntenic with *E. coli* O157 : H7 strain Sakai and each other (Fig. S1). Comparison of the two complete genomes with the NCBI non-redundant database (Table S2) and each other revealed a common architecture consisting of a single bacterial chromosome and three plasmids (Fig. 2). The smallest plasmid was a ~2 kb cryptic plasmid that had been detected in unpublished *E. coli* draft genomes and described fully as SpC in *S. sonnei* Ss046 [32]. The second and larger plasmid shared 100 % coverage and 99 % nucleotide identity with p09EL50, a plasmid detected in an STEC O104:H4 isolate recovered from a patient with diarrhoea in the Republic of Georgia in 2009 [33]. Finally, there was a large resistance plasmid related to (83 % coverage, 99 % identity) pHUSEC41-1, a plasmid recovered from an STEC O104:H4 isolate from a patient with haemolytic-uraemic syndrome in Germany in 2001 [34], and that shared a similar level of similarity (75 % coverage, 99 % identity) to a resistance plasmid from a MSM-associated sublineage of *S. sonnei* circulating in UK MSM (MSMA sublineage 3 in [6]) over a similar time frame (Fig. 3).

### Virulence determinants

To investigate why an STEC serotype that is normally rare in the UK caused an outbreak, the presence of virulence determinants was characterized for all isolates (Table S1, shown in detail for isolate 23168 in Table 2). Further to this, the complete genome of isolate 23169 was also explored for the presence of known STEC PAIs and the *stx* prophage. This revealed the existence of seven ubiquitous (specifically *capU*, *gad*, *iha*, *sigA, stx1*, *stx1a*, *stx1b*) virulence determinants and seven non-ubiquitous virulence determinants among the 36 STEC O117 : H7 isolates (none of which were enriched in the outbreak clade; Table S1, Fig. S2). Outbreak isolate 23 169 did not carry the LEE-PAI or SE-PAI, but did possess some elements of the recently described modular LAA-PAI. Specifically, positions 3928350–3935630 of the 23169 genome (contiguous sequence 1) shared 97 % identity with part of module 1, inclusive of genes encoding the AtoSC two-component system [35], but not the key virulence genes *sisA* or *hes*. Similarly, positions 1502495–1517274 shared 93 % identity with part of module 4 of LAA-PAI, inclusive of ORFs 66–80 (including the known virulence factor antigen 43 [36]) with the synteny being interrupted at ORF 69 by the insertion of a transposase (IS*66*) in isolate 23169 (Fig. S3). The Stx-encoding prophage was found in positions 3711427–3799540, with its closest described relative being a novel *stx1a* phage Ss-VASD, recently isolated from *S. sonnei* taken from human patients with diarrhoea who had recently travelled to Mexico [37]. However, comparative genomic analysis revealed that the prophage and Ss-VASD had significant dissimilarity (56 % fragmented coverage with 96 % nucleotide identity). In fact, pairwise alignment of the prophage and phage sequences resulted in an overall nucleotide identity of 61.6 %, suggesting that the prophage is a novel species according to current guidelines [38]. Accordingly, this T12011virus-like prophage was putatively named ‘*Escherichia* phage GER2’ and was deposited separately in GenBank under accession number MG710528, with annotation information from the phast pipeline.

**Table 2.** Virulence determinants and locations found in isolate 23168

Virulence factor	Contiguous sequence	Position	Protein function	**Accession no.**
*capU*	ERS1746629|SC|contig000001	1543785–1544873	Hexosyltransferase homologue	CP003034
*gad*	ERS1746629|SC|contig000001	2456206–2457606	Glutamate decarboxylase	CP001671
*iha*	ERS1746629|SC|contig000001	3055028–3057118	Adherence protein	AE005174
*sigA*	ERS1746629|SC|contig000001	3077450–3081307	*Shigella* IgA-like protease homologue	CP000038
*stx1a*	ERS1746629|SC|contig000001	3751577–3752525	Shiga toxin 1, subunit A, variant a	EF079675
*stx1b*	ERS1746629|SC|contig000001	3752535–3752804	Shiga toxin 1, subunit B, variant a	AM230663
*gad*	ERS1746629|SC|contig000001	4771849–4773249	Glutamate decarboxylase	CP001671
*stx1*	ERS1746629|SC|contig000001	3751577–3752804	*Shigella dysenteriae* 3818T	M19437
*astA*	ERS1746629|SC|contig000003	22 974–23090	EAST-1 heat-stable toxin	AB042002
*cba*	ERS1746629|SC|contig000003	2916–3854	Colicin B	FJ664721
*senB*	ERS1746629|SC|contig000003	31 963–33138	Plasmid-encoded enterotoxin	CP000038
*cba*	ERS1746629|SC|contig000003	9589–10527	Colicin B	FJ664721
*celb*	ERS1746629|SC|contig000012	5441–5584	Endonuclease colicin E2	AF540491

### AMR determinants

The draft genomes of all 36 isolates and complete genomes of the two SM-RT-sequenced isolates were examined for genes and mutations known to confer reduced susceptibility to antimicrobials. The isolates were found to have numerous genetic determinants of resistance against a variety of antimicrobial classes (Table 2), with all but two isolates containing genes consistent with a multidrug-resistance phenotype (resistance against ≥3 antimicrobial classes; Table S1, Fig. S2). Resistance against azithromycin and fluoroquinolones was further investigated because of the recent findings of the importance of these resistance profiles to STEIs in MSM [39]. Four of the six epidemiologically confirmed outbreak isolates contained the azithromycin-resistance gene *mphA* and all four *mphA*-containing outbreak isolates were confirmed to have high-level phenotypic resistance against azithromycin (i.e. MICs of 128–>256 mg ml^−1^, Table S1). The presence of azithromycin-resistance genes was enriched in the outbreak clade relative to the remainder of the tree (5 of 10 isolates, compared with 4 of 26 isolates) though this association fell short of traditional significance measures (*P*=0.0794 rather than *P*<0.05, Fisher’s exact test). Although both isolates for which complete genomes were sequenced contained the *mphA* gene in their draft genomes (and had phenotypic azithromycin resistance confirmed) only one of the two (23168) had the *mphA* gene in the complete genome, suggesting it may have been lost during re-culture. All ten isolates in the outbreak clade had a mutation in the quinolone-resistance-determining region of *gyrA* (S83L), which is associated with reduced susceptibility against fluoroquinolones [40], though this was also common among non-outbreak clade isolates (Table 3, Fig. S2). Notably, five of the ten resistance genes in isolate 23168 were carried on the large pHUSEC41-1-like resistance plasmid (Fig. 3).

**Table 3.** Genetic determinants of AMR among the isolates

Antimicrobial class	Proportion of isolates (%)	Genetic determinants (no. of isolates out of 36)
Aminoglycosides	42	*aadA1* (9), *aadA2* (5), *aadA5* (1)
β-Lactams	47	*bla*_TEM-1B_ (14), *bla*_TEM-1C_ (3)
Trimethoprim	64	*dfrA1* (12), *dfrA12* (5), *dfrA14* (1), *dfrA* (17), *dfrA5* (15)
Macrolides	25	*mphA* (9), *ermB* (1)
Fluoroquinolones	58	*gyrA* S83L mutation (21), *qnrs1* (1)
Streptomycin	64	*strA* and *strB* (23)
Sulphonamide	94	*sul1* (14), *sul2* (29)
Tetracycline	58	*tet(A)* (18), *tet(B)* (3)

## Discussion

Combining the evolutionary analysis of outbreak and contextual STEC O117 : H7 isolates with traditional epidemiological markers yielded insights into the possible origins, and potentially ongoing nature of the outbreak. In addition to the six isolates known to belong to the outbreak, the encompassing outbreak clade included a further four isolates, three of which were recovered from patients recently returned from travel to Latin America in 2010, 2013 and 2016. This suggests that the outbreak strain may have originated in, and continues to circulate in, Latin America. Though more work is needed to investigate this link, this mirrors the findings for MSM-associated *Shigella flexneri* 3a that emerged in the UK, which was also potentially imported from Latin America [4]. The fourth non-epidemiologically confirmed isolate in the outbreak clade was from an adult male whose infection was contracted in the UK (London) in 2017, suggesting the outbreak strain may still be circulating in the UK MSM community (though this speculation is based solely on the patient demographic characteristics, as no epidemiological follow-up of this case was performed).

The evolutionary analysis also provided genomic evidence for chronic infection with STEC O117 : H7. Specifically, two isolates (outside of the outbreak clade) from the same patient taken 4 months apart were indistinguishable in this analysis (identical across the 3694 variant sites used for phylogenetic inference, no virulence or AMR determinant content differences). This supports the epidemiological investigation of the outbreak, where symptoms of ≥1 month were reported [7], and other studies of STEC O117 : H7 that have also reported repeated isolation for up to 96 weeks of the same serotype in chronically symptomatic persons [11]. Again, this genomic evidence of chronic infection mirrors observations in *S. flexneri* 3a among UK MSM where isolates serially isolated from patients ≤154 days apart were genetically similar (0–4 SNPs) [4]. Although clinical investigations are urgently needed to accurately determine the duration of infection and clinical presentation (e.g. asymptomatic, intermittently symptomatic, chronically symptomatic) of STEIs in MSM, these findings indicate that multiple STEIs are capable of chronic (≥4 months duration) infection.

Analysis of the accessory genome of the outbreak isolate indicated likely horizontal gene flow between pathogenic *E. coli* and shigellae. This was supported by the presence of plasmid p09EL50 in the outbreak isolate; a plasmid originally described in a 2009 STEC O104 : H4 strain from the Republic of Georgia [33]. Similarly, the pHUSEC41-like large resistance plasmid in the outbreak strain was most closely related with a plasmid isolated from a sublineage of *S. sonnei* (MSM-associated sublineage 3) that was circulating in UK MSM over a similar timeframe to the 2014 STEC outbreak, providing a plausible opportunity for direct or indirect genetic exchange between these two pathogen subtypes. The finding of shared AMR determinants among distinct STEIs in the MSM community complements results from a recent WGSA study of overlapping *Shigella* epidemics in UK MSM where a resistance plasmid, pKSR100, was shared among five co-circulating polyphyletic *Shigella* sublineages (three of *S. sonnei*, one of *S. flexneri* 2a and one *S. flexneri* 3a) [5, 6].

To explore a possible pathogenic basis for the emergence of this rare STEC serotype in MSM, outbreak isolates were investigated for the presence of virulence determinants. Virulence determinants associated with more severe disease presentations, including Stx2a and the LEE-PAI, were not present in the outbreak clade. Increasing evidence suggests that LEE-negative STEC may have common virulence determinants that contribute to their clinical presentation such as the SE- and LAA-PAIs [41, 42]. Although partial modules of the recently described LAA-PAI were present, the regions of similarity did not contain many of the key virulence determinants, and the SE-PAI was absent. The outbreak clade did contain the Shiga-toxin-encoding genes *stx1a* and *stx1b* on a novel prophage most closely related with an *stx1a* phage isolated from *S. sonnei* from human patients in Mexico [37], and also contained the virulence factor *sigA*. However, these key virulence determinants typically associated with *Shigella* (i.e. *stx1a, stx1b* and *sigA*) were also ubiquitous among the contextual isolates. To summarize then, virulence determinants were not markedly different between outbreak and contextual isolates, so were not likely to have contributed to the aberrant emergence of this rare serotype in MSM.

Outbreak and contextual isolates were distinguishable, however, through the enrichment of genes encoding azithromycin resistance among isolates in the outbreak clade, highlighting the increasingly apparent role of AMR in epidemics of STEIs in MSM. The three subtypes of *Shigella* causing outbreaks among UK MSM were also associated with azithromycin resistance relative to the contextual isolates in those studies [4, 6]. This common association of azithromycin resistance among four different STEIs in UK MSM confirms the importance of this resistance phenotype in driving STEI emergence in this patient group. Although no specific studies have evaluated relative antimicrobial usage patterns between STEI-affected MSM and other patient groups, treatment reviews have shown that antimicrobial use is frequent in STEI-affected MSM [43, 44] and that many patients have recent or co-morbid infection with other bacterial STIs, many of which are treated with azithromycin [4, 12]. These two features combined would plausibly create sustained selective pressure for azithromycin resistance, particularly for pathogens where the infection window is chronic. And, the chronic infection window also facilitates ample opportunity for horizontal genetic exchange between STEI pathogens in MSM, either directly or indirectly via the microbiota, which may act as a reservoir for AMR in these patients.

These results have implications for public health practice. Outbreaks of enteric illnesses are not typically investigated for association with sexual activity in MSM (though this is changing in the UK consequent to the recent epidemics). The continued finding of STEI emergence associated with azithromycin resistance indicates that it may be useful to investigate and manage azithromycin-resistant enteric illness outbreaks as being potentially associated with sexual transmission. Furthermore, the tendency of STEIs in MSM to be resistant to azithromycin, which is not necessarily the primary treatment for enteric illness, suggests that treatment for other illnesses, including non-enteric bacterial STIs, may be creating a sustained selection pressure for resistance in other pathogens. This selection pressure can then drive pathogen evolution through horizontal gene transfer and potentially cause unexpected changes in epidemiology, such as the emergence of a rare serotype. To work toward a public-health framework where such rapid changes in epidemiology are de-mystified, we must continue to scrutinize pathogens at the genomic level, characterize gene flow among different bacterial populations, and integrate this information with detailed epidemiological data.

## Data bibliography

1. Public Health England, Sequence Read Archive, Bioproject number: PRJNA315192 (2017).
2. Baker KS. Figshare. doi: 10.6084/m9.figshare.6272216 (2018).

## Supplementary Data

Supplementary File 1

## Supplementary Data

Supplementary File 2
