# Supplementary material for: An outbreak of a rare Shiga-toxin-producing Escherichia coli serotype (O117:H7) among men who have sex with men
Source: Microb Genom. 2018 May 21;4(7):e000181. doi: 10.1099/mgen.0.000181 (PMC6113874; doi:10.1099/mgen.0.000181)
Supplement: Supplementary File 1 [file mgen-4-181-s001.pdf]

Supplementary Information

An outbreak of a rare STEC serotype (O117:H7) among men who have sex with men

Kate S Baker, Timothy J Dallman, Nicholas R Thomson, Claire Jenkins

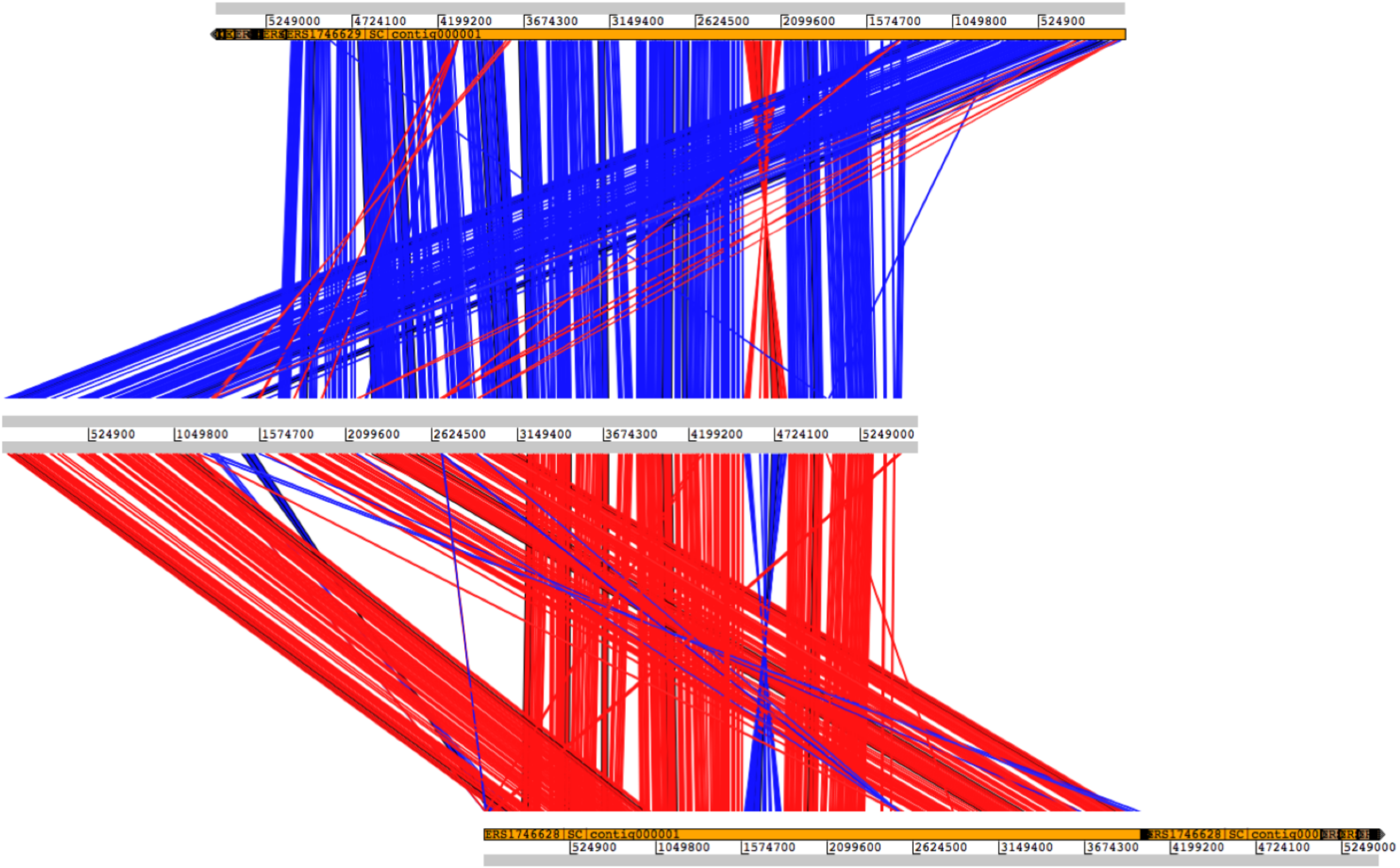

Figure S1. Comparison of outbreak genomes with *E. coli* O157:H7. Ordered contiguous sequences for outbreak isolate 23169 (upper, flipped) and 23168 (lower) are shown compared with *E. coli* O157:H7 strain Sakai (middle, NCBI accession number: NC_002695.1). Intervening red and blue bars show regions of synteny, filtered by a lower BLAST score of 6000. For the two outbreak isolates, the contiguous sequence boundaries are shown by overlaid yellow genome annotations.

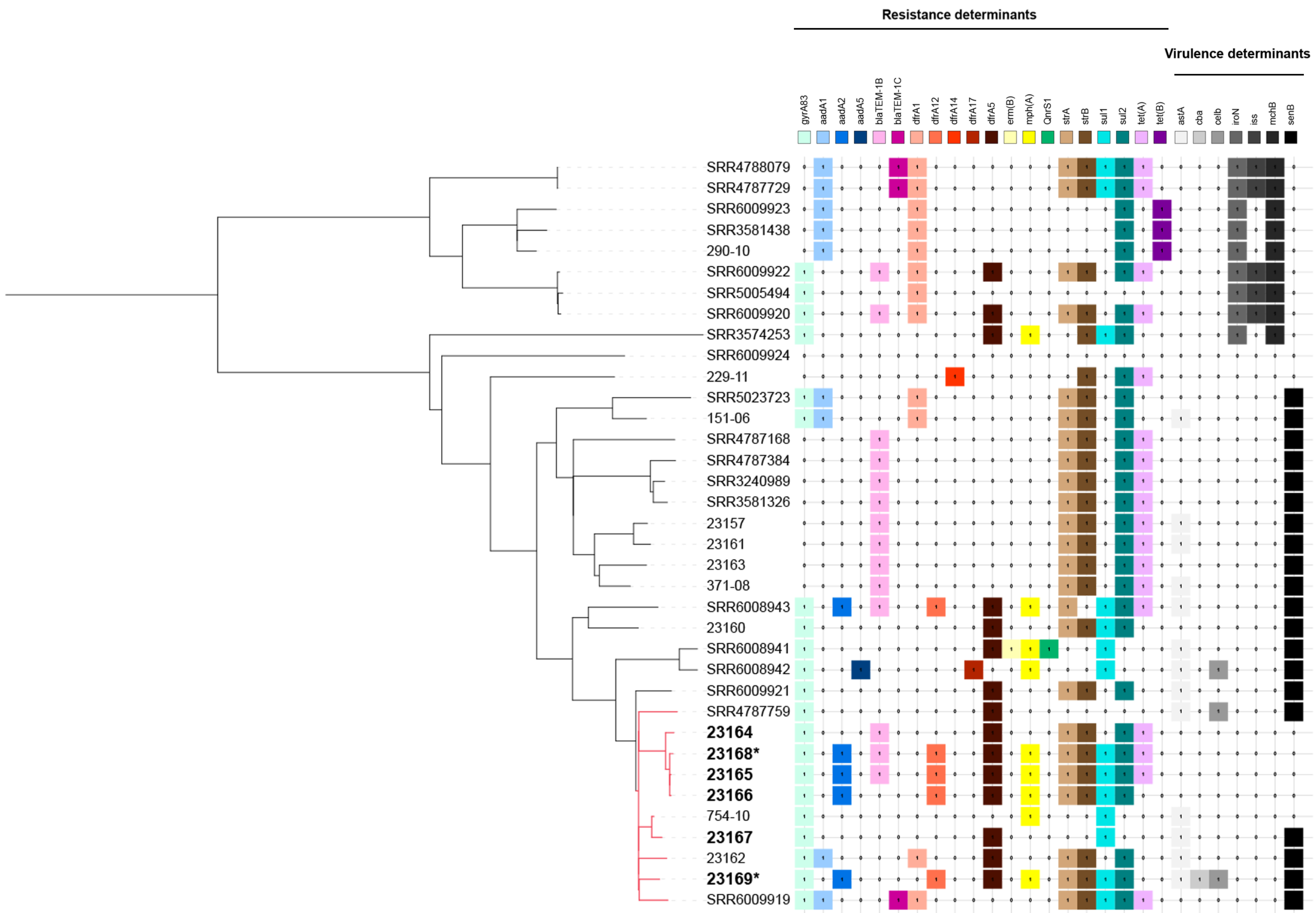

Figure S2. Presence of antimicrobial resistance and virulence determinants across *E. coli* O117:H7'. The phylogenetic tree of isolates (from Fig. 1) is shown with the adjacent tracks showing the presence of various antimicrobial resistance and virulence determinants across the isolates.

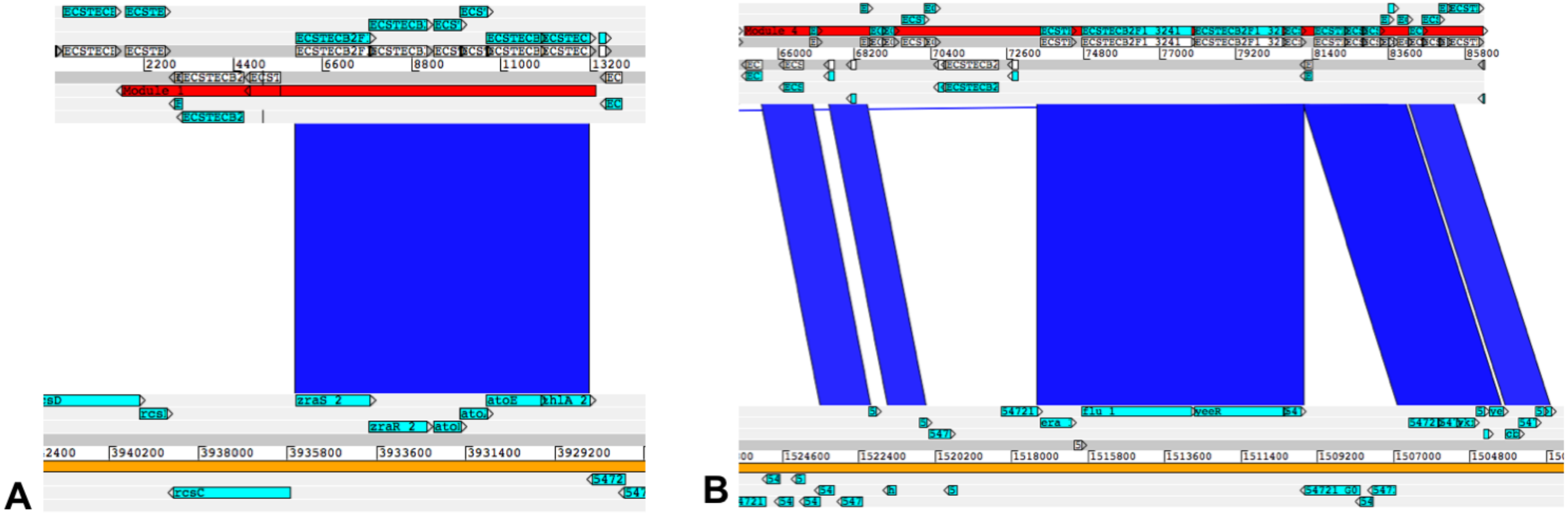

Figure S3. Comparison of outbreak isolate 23169 with LAA-PAI. The genome of outbreak isolate 23169 is shown (lower) compared with LAA-PAI (upper) modules 1 (A) and 4 (B). Intervening blue and red bars indicate regions of synteny. Annotations of genome features and CDS are shown in white and blue for each genome, while module boundaries are shown in red.
